# Tuberculosis in the ICU: a retrospective cohort study

**DOI:** 10.1186/cc14167

**Published:** 2015-03-16

**Authors:** R Duro, P Figueiredo, A Ferreira, S Xerinda, C Lima Alves, L Santos, A Sarmento

**Affiliations:** 1 Centro Hospitalar de São João/Faculty of Medicine University of Porto, Portugal

## Introduction

To describe the characteristics of the patients with tuberculosis (TB) requiring intensive care and to identify the factors associated with in-hospital mortality in an ICU in Portugal.

## Methods

A retrospective cohort study between January 2007 and July 2014 of all patients with TB admitted to the ICU of the Infectious Diseases Department of Centro Hospitalar de São João. Comorbid diagnoses, clinical features, radiological and laboratory investigations and outcomes were reviewed. The primary outcome was the in-hospital mortality. A univariate analysis was performed to identify risk factors for death.

## Results

During the study period, 40 patients with TB were admitted to the ICU; 75% male and median age of 52 years (IQR 37.5 to 62.8). Overall, 22 (55%) patients died in the hospital, of whom 16 (40%) died in the ICU. Comorbid illness was identified in 32 (80%) patients, with HIV infection being the most common, present in 15 (37.5%) patients. The main reason for ICU admission was respiratory failure (70%), followed by sepsis/septic shock (22.5%). Twenty-eight (70%) patients had isolated pulmonary disease, four (10%) had isolated extrapulmonary disease and eight (20%) had association of pulmonary and extrapulmonary disease. Mycobacterial cultures were positive in 31 (77.5%) patients; three patients presented monoresistant strains. Twenty-nine (72.5%) patients required mechanical ventilation and 21 (52.5%) required vasopressor infusion in the ICU; two patients were treated with ECMO. Thirty-four (85%) patients received antituberculosis therapy. The median length of stay was 11.5 (IQR 3.25 to 28.5) days in the ICU and 40.5 (IQR 21.0 to 62.8) days in the hospital. The presence of at least one comorbidity, smoking, age, sepsis/septic shock on admission, high SAPS II and APACHE II score, positive direct examination and PCR in respiratory samples, the need for mechanical ventilation or vasopressor infusion were significantly associated with mortality (*P *< 0.05). There was no association between mortality and HIV status, site of TB disease, concomitant acute disease or development of hospital infections.

## Conclusion

In this cohort we found a high mortality rate in the TB patients requiring intensive care. The risk factors for mortality due to severe TB are mainly related to the severity of organ failure, patient characteristics and burden of disease and not to HIV status or site of TB disease.

